# Editorial: New challenges in animal welfare

**DOI:** 10.3389/fvets.2023.1178950

**Published:** 2023-03-21

**Authors:** Rodrigo Muiño, Joaquin Hernández, Jose Luis Benedito, Cristina Castillo

**Affiliations:** Department of Animal Pathology, Faculty of Veterinary Medicine, University of Santiago de Compostela, Lugo, Spain

**Keywords:** transport, regulations, welfare, animal health, equine and dogs

In recent decades, society's awareness of animal welfare has increased considerably, and among others, the main demand of consumers in the European market is the improvement of animal conditions. This interest has provoked direct consequences for the farms, which must visibly and transparently improve the management of the animals toward society. Then, animal welfare legislation has made considerable progress in European countries. It has developed a framework for protecting farm animals that ensures that they are housed and transported in conditions that do not involve mistreatment or cause them pain or suffering.^1^,^2^ In this way, people will better accept the farm production systems. But there are still hurdles to overcome, like current regulations based on environmental or resource-related actions. There is minimal interest in implementing measures on the animal itself (1).

This Research Topic aims to provide scientific information to help improve knowledge about animal welfare. We understand that there are many points to improve in this area, and one of the most important and complex is to provide better objective measurement of animal welfare, to find valid and reliable indicators of the state of the animals, including the different criteria of animal welfare (physical health and emotional state).

In this Research Topic, four articles deal with the above-mentioned aspects. The tests have been conducted on two animal species, canine, and equine. The two studies published in this Research Topic on the canine species have in common the originality of their starting hypotheses and their enormous contribution to animal welfare in this species.

In line with improving the welfare of our pets, a second research paper was published, considering social networks, which are widely used in our society, explored for the first time the sentiment expressed by four different sectors in the context of animal welfare: Personal (for instance the public in the UK and the Republic of Ireland), press, state (for instance. the Government, Police, and the NHS) and other (i.e., charities, social enterprises, research organizations, charities, and businesses).

Discussions focused on issues such as separation anxiety, dog theft, and the meat trade. The sectors that most influence public perception has mainly been the press and the state. Therefore, the messages used by public authorities and the press in social media must be as accurate and responsible as necessary since they are the ones that most influence public opinion in society on the relationship between humans and animals.

The two studies published on the equine species have in common that both provide scientific data that should be considered in future modifications of animal welfare regulations for transport, although the current European regulation (EC 1/2005) only considers environmental considerations or own resources to avoid suffering or pain during the transport of animals. The initial aim of this research article was to develop and validate a broken (handled)/not broken (not handled) (BUT) test to identify unbroken horses, although two articles in this issue go a step further by considering factors that also affect the animal itself, such as through spacing (Baumgartner et al.) or transport of unbroken horses (Riva et al.), whose regulations are very restrictive although they do not provide adequate tools to identify unbroken horses. The main objective of this research article was to develop and validate a tame/non-tamed horse (BUT) test to assess whether a horse is tamed or not. To evaluate this test, they used 100 healthy Italian heavy draft horses, and continuously videotaped their behavior, which was evaluated by three blind observers. This study showed that unmanipulated horses were statistically significantly more likely to show a flight reaction when approached by a person than manipulated horses. This trial gave validity to the BUT test for classifying horses as broken or unbroken. It also pointed out that it would be highly recommended to perform the test before loading the horses to protect their welfare during transport.

Baumgartner et al. described the importance of an adequate animal-to-feeder ratio (AFR) in time-controlled hay feeders for horses. These feeders have no partitions and are more likely to cause injuries between horses when used compared to feeders with separations or transporter-controlled feeding stations. When studying the behavior of horses using time-controlled hay bunkers, he concluded that they had to have at least three times as many feeders as horses to ensure that neighboring horses could maintain their distance from each other. And questioning the previous recommendations, that only 20% more feeders are available than in free stall systems with time-controlled hay bunkers.

In summary, the results of the studies published in this Research Topic of the journal Frontiers Veterinary Science provide new relevant data on animal welfare in the canine and equine species. Together with other previously published work in this area of research, they contribute to improving the welfare of our domestic animals, and to provide a fairer and more responsible society for animals.

## Author contributions

All the editors have reviewed the manuscripts and the comments of the various reviewers. All authors contributed to the article and approved the submitted version.

## Footnotes

^1^The welfare of animals during transport (details for horses, pigs, sheep and cattle). Report of the scientific commitee on Animal Health and Animal Welfare. Available online at: https://food.ec.europa.eu/system/files/2020-12/sci-com_scah_out71_en.pdf.

^2^Council Regulation (EC) No 1/2005 of 22 December 2004 on the protection of animals during transport and related operations. Available online at: https://eur-lex.europa.eu/legal-content/EN/ALL/?uri=CELEX:32005R0001.
